# No Effect of Interstimulus Interval on Acoustic Reflex Thresholds

**DOI:** 10.1177/2331216519874165

**Published:** 2019-09-13

**Authors:** Hannah Guest, Kevin J. Munro, Samuel Couth, Rebecca E. Millman, Garreth Prendergast, Karolina Kluk, Carlyn Murray, Chris Plack

**Affiliations:** 1 Manchester Centre for Audiology and Deafness, Manchester Academic Health Science Centre, University of Manchester, UK; 2 Manchester University NHS Foundation Trust, UK; 3 Department of Psychology, Lancaster University, UK

**Keywords:** acoustic reflex, middle-ear-muscle reflex, auditory nerve, retrocochlear disorder, cochlear synaptopathy

## Abstract

The acoustic reflex (AR), a longstanding component of the audiological test battery, has received renewed attention in the context of noise-induced cochlear synaptopathy—the destruction of synapses between inner hair cells and auditory nerve fibers. Noninvasive proxy measures of synaptopathy are widely sought, and AR thresholds (ARTs) correlate closely with synaptic survival in rodents. However, measurement in humans at high stimulus frequencies—likely important when testing for noise-induced pathology—can be challenging; reflexes at 4 kHz are frequently absent or occur only at high stimulus levels, even in young people with clinically normal audiograms. This phenomenon may partly reflect differences across stimulus frequency in the temporal characteristics of the response; later onset of the response, earlier onset of adaptation, and higher rate of adaptation have been observed at 4 kHz than at 1 kHz. One temporal aspect of the response that has received little attention is the interstimulus interval (ISI); inadequate duration of ISI might lead to incomplete recovery of the response between successive presentations and consequent response fatigue. This research aimed to test for effects of ISI on ARTs in normally hearing young humans, measured at 1 and 4 kHz. Contrary to our hypotheses, increasing ISIs from 2.5 to 8.5 s did not reduce ART level, nor raise ART reliability. Results confirm that clinically measured ARTs—including those at 4 kHz—can exhibit excellent reliability and that relatively short (2.5 s) ISIs are adequate for the measurement of sensitive and reliable ARTs.

## Introduction

 The acoustic reflex (AR)—also known as the acoustic middle-ear-muscle reflex or stapedius reflex—is a reflexive contraction of the stapedius muscle in response to high-intensity acoustic stimuli, leading to changes in the acoustic immittance characteristics of the middle ear. Measurement of AR thresholds (ARTs) has been a standard element of the audiological test battery, valuable especially in the diagnosis of middle-ear and retrocochlear disorders. Recently, renewed interest in the AR has been provoked by the discovery of noise-induced cochlear synaptopathy—the loss of synapses between cochlear inner hair cells and auditory nerve (AN) fibers, which can occur without widespread hair cell loss or permanent threshold elevation (Liberman & Kujawa, 2017). Detection of synaptopathy in living humans requires noninvasive metrics of AN function, and the AR may be of value. In a mouse model of synaptopathy, AR measures correlated more closely with synaptic survival than auditory-brainstem-response measures, with ARTs proving superior to AR amplitude in predicting synapse counts (Valero, Hancock, Maison, & Liberman, 2018). In investigations of tinnitus-related synaptopathy, both AR amplitude (Wojtczak, Beim, & Oxenham, 2017) and ARTs (Guest, Munro, & Plack, 2019a) have been adopted as proxy measures of AN function.

A potential difficulty in applying ARTs to the detection of noise-induced synaptopathy—and in ART measurement in general–is that measurement at high stimulus frequencies is not always straightforward. Early noise-induced auditory damage tends to manifest in the 3 to 6 kHz region (Coles, Lutman, & Buffin, 2000), and if this pattern extends to synaptopathy, AR measurement focusing on high-frequency AN fibers may be important. Yet 4 kHz ARTs are often elevated or absent, even in young people with normal audiograms (Gelfand, 2009; Hunter, 2013; Maia & Russo, 2008). Given the strong relation observed between ARTs and synaptopathy in the mouse model (Valero et al., 2018), it is tempting to regard widespread absence of 4 kHz reflexes as a potential indicator of prevalent synaptopathy in the normal-hearing human population.

However, it remains possible that absent responses at 4 kHz are attributable to factors other than the loss of functional AN fibers. In particular, dynamic characteristics of the response at 4 kHz are known to differ from those at lower frequencies. Jerger, Oliver, and Stach (1986) observed that higher stimulus frequencies are associated with increased AR onset latency. Wilson, Steckler, Jones, and Margolis (1978) demonstrated that, at 4 kHz, adaptation begins almost immediately after stimulus onset and that the higher the stimulus frequency, the higher the rate of adaptation. It is plausible that ART elevation at 4 kHz might be related in some way to across-frequency differences in the dynamic characteristics of the response. Indeed, it has been suggested that rapid adaptation might underlie the high prevalence of elevated 4 kHz ARTs in young, normally hearing people (Gelfand, 2009).

One dynamic aspect of the AR that has received little attention—at least in relation to stimulus frequency—is its offset characteristic: the manner in which the response is affected by the silent interval between successive stimuli. Yet findings in a number of human cohorts suggest that an inadequate interstimulus interval (ISI) might reduce AR amplitude via fatigue effects. Borg and Odman (1979) demonstrated that the AR exhibits slow recovery after decay-producing stimulation; for one participant, a silent interval of 10 s was required to allow full recovery. Possible effects of ISI on ARs elicited by much briefer (500 ms) tones were investigated by Jerger, Mauldin, and Lewis (1977; ISIs = 0.75–5 s) and Jerger and Oliver (1987; ISIs = 1.5–9 s); results indicated that longer ISIs might enhance AR amplitudes in older subjects, though not in the young. However, both sets of observations were confined exclusively to a stimulus frequency of 1 kHz; it is plausible that the effects of ISI at higher frequencies might be more pronounced, especially given the well-established finding of rapid AR decay at 4 kHz (Fowler & Wilson, 1984; Wilson et al., 1978). To our knowledge, potential effects of ISI on the AR at high frequencies are untested.

To address this gap in the literature, we conducted two studies to assess the effects of ISI on ARTs, measured using widely available clinical recording equipment. We hypothesized that lengthening ISIs would lead to lower 4 kHz ARTs, with no such effect evident at 1 kHz. A secondary hypothesis was that lengthening ISIs would enhance ART test–retest reliability at 4 kHz.

## Material and Methods

### Overview

Two consecutive studies, with near-identical methods, were conducted by two researchers with differing experience in ART measurement, each assessing potential effects of ISI on ART level and reliability. For Study 1 (conducted by the lead author), a detailed research protocol was registered prior to data collection via the Open Science Framework (registered in Pre-Reg Challenge format, publicly available at https://osf.io/4n7s3/); all subsequent procedures and analyses conformed strictly to the protocol, with the exception of the sample size (see Participants section) and the selection of an appropriate nonparametric test for use with non-normally distributed data (see Analysis section). For Study 2 (conducted by a postgraduate student), no protocol was registered, but methods were largely shared with Study 1; all deviations are described in the relevant subsections of the Methods section. Where no reference is made to a specific study, the reader may assume that the reported methods were shared by both studies. We reasoned that parallel investigation of our research questions by two independent researchers would—if results were consistent—justify greater confidence in the findings and allow generalization of our conclusions to both inexperienced and relatively experienced ART practitioners.

For Study 1, participants attended two test sessions, separated by at least 1 day and fewer than 30 days. In Study 2, participants attended a single test session, with test and retest thresholds obtained by simply removing and replacing the probe tip. ARTs were measured monaurally; the same ear was tested at the two sessions. Choice of test ear was determined by various factors, for example, presence of wax in the ear canal, locations of piercings, participant preference, or recording difficulties affecting a single ear; if none applied, the right ear was selected. During data collection and analysis, care was taken to ensure that each researcher was blind to the findings of the other. All procedures were approved by the Human Communication, Development and Hearing Ethics Panel at the University of Manchester.

### Participants

Participants aged 18 to 30 years were recruited via advertising on the University of Manchester website. Participants reported no history of middle-ear surgery, hearing loss, neurological disorder, ototoxic exposure, or head trauma. All test ears were required to exhibit normal otoscopic findings, normal tympanometric results (compliance 0.3–1.6 cm^3^, pressure −50 to + 50 daPa), normal pure tone audiometric thresholds (≤20 dB HL at 0.25–8 kHz), and measurable ARTs at both test frequencies. If only one ear conformed to the above criteria, then that ear was selected for testing; if neither ear conformed, then the individual was excluded from participation. Sample size for Study 1 (*n* = 24) deviated from that specified in the study protocol (*n* = 40), due to an error in the a priori power analysis, in which the expected standard deviation of difference was markedly overestimated (3.4 dB, cf. 2.6 dB post hoc). Consequently, data collection was drawn to a close once the post hoc *power* exceeded the target explicitly stated in our registered protocol (80% power to detect a 2 dB change in ART; α = 0.01, two-tailed). For Study 2, the sample size was 36. As both studies recruited from the same source, the cohorts overlapped somewhat: Seven participants took part in both studies; the remainder of each sample was independent. The Study 1 cohort had a mean age of 22.8 years (*SD* = 2.7 years) and was composed of 20 females and 4 males. The Study 2 cohort had a mean age of 23.3 years (*SD* = 3.0 years) and was composed of 24 females and 12 males.

### Pure Tone Audiometry

Participants were seated in double-walled sound-attenuating booth and received pure tone stimuli via a GSI Arrow audiometer coupled to TDH-39 supra-aural headphones, calibrated to BS EN 60645-1 (British Standards Institution, 2017). Air-conduction thresholds at 0.25, 0.5, 1, 2, 4, and 8 kHz were obtained from both ears in accordance with the recommended procedures of the British Society of Audiology (2011).

### AR Thresholds

ARTs were measured monaurally using a GSI Tympstar diagnostic middle-ear analyzer, calibrated to BS EN 60645-5 (British Standards Institution, 2005). The probe was encompassed in a Grason KR-Series clinical ear tip and delivered a 226 Hz probe tone. Stimuli were ipsilateral pulsed pure tones at 1 and 4 kHz with a duration of 1.5 s. Prior to measurement, the participant was counseled on the following: (a) basic setup and purpose of the test; (b) sound quality of the stimuli; (c) timing of the stimuli; (d) timing of the response periods; (e) importance of artifact-free response periods; (f) possible sources of measurement artifact (e.g., swallowing, movement, and vocalization); (g) test duration; (h) importance of avoiding loudness discomfort; and (i) verbal and nonverbal means of halting the test.

Reflex thresholds were determined by observing changes in middle-ear compliance following presentation of the stimuli. A reflex response was defined as a reduction in compliance of 0.02 ml or greater with appropriate morphology and no evidence of significant measurement artifact. If significant measurement artifact was observed during the response period, the presentation was repeated. For each threshold measurement, stimulus level commenced at 70 dB HL and ascended in 5 dB steps until a response was observed. Stimulus level then decreased in 5 dB steps until a response was no longer observed and subsequently ascended in 2 dB steps until threshold was obtained. Threshold was defined as the lowest stimulus level at which three clear responses were observed over the course of three, four, or five artifact-free presentations.

ISI was controlled by the researcher, guided by a smartphone metronome app set to indicate intervals of 1 s (JSplash Apps, 2018). For the *short* ISI, a duration of 2.5 s was selected, since this was the minimum ISI consistently feasible with our clinical recording equipment, which often required multiple button pushes between one presentation and the next. For the *long* ISI, a duration of 8.5 s was selected, resembling the ISI shown to reduce ARTs in the elderly by Jerger and Oliver (1987). Because the stimuli had a duration of 1.5 s, the 2.5 s ISI was achieved by implementing a stimulus onset asynchrony (the interval between the onset of one stimulus and the next) of 4 s; the 8.5 s ISI was achieved by implementing a stimulus onset asynchrony of 10 s.

Eight ARTs were measured to assess the effects of ISI (2.5 or 8.5 s) at two stimulus frequencies (1 and 4 kHz), with two repetitions of each condition (test and retest) to assess reliability. In Study 1, the test and retest ART measurements were conducted at different test sessions; in Study 2, retesting was conducted after simply removing and replacing the probe tip. The order of presentation of the two ISIs was counterbalanced to prevent the influence of order effects on observed results. In Study 1, odd-numbered participants received stimuli with short (2.5 s) ISIs first, while the remainder received stimuli with long (8.5 s) ISIs first; in Study 2, choice of order was quasi-random rather than strictly alternating, with 50% of participants ultimately receiving each presentation order. Order of stimulus frequency was not counterbalanced in either study. Presentation order was identical between test and retest for each participant.

### Analysis

All statistical analyses were conducted in R (R Core Team, 2015). To answer the main research question, ARTs were averaged across the two repetitions (test and retest), yielding four threshold averages (1 kHz, 2.5 s ISI; 1 kHz, 8.5 s ISI; 4 kHz, 2.5 s ISI; and 4 kHz, 8.5 s ISI). Effects of ISI on average threshold (at both 1 and 4 kHz) were assessed via paired *t* test or paired sample Wilcoxon test, as appropriate to the data distribution. This approach allowed analysis of nonnormally distributed thresholds obtained at 4 kHz and was—despite the studies’ multifactorial design—suitable for addressing our research question, since a main effect of ISI was not hypothesized; rather, a simple effect was anticipated, that is, an effect of one independent variable (ISI) *within a single level* of a second independent variable (stimulus frequency).

Test–retest reliability of the thresholds (at the two frequencies and ISIs) was assessed by computation of four intraclass correlation coefficients (ICCs), which express the ratio of true variance to the sum of true variance and error variance. The specific form of ICC employed was a one-way, random-effects, single-rater ICC, denoted ICC(1,1) by Shrout and Fleiss (1979). The use of a one-way, random-effects model yields a more conservative estimate of reliability than the other forms of ICC and effectively regards each test session for each participant as a separate random judge sampled from the population, acknowledging that different test sessions involve differences in time of day, participant state, ear-tip position, and so on. In the Results and Discussion sections, the descriptors of reliability (*moderate*, *good*, etc.) follow the conventions of Koo and Li (2016).

## Results

### Effects of ISI on ART Level

**Figure 1.**
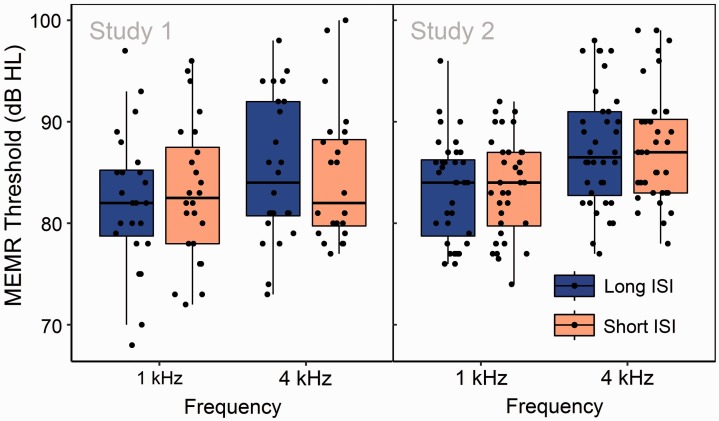
Distributions of ARTs at 1 and 4 kHz, obtained using short (2.5 s) and long (8.5 s) ISIs. Points correspond to individual participants, upper and lower hinges to the first and third quartiles, upper whiskers to the highest value within 1.5 × IQR of the upper hinge (where IQR is the interquartile range), and lower whiskers to the lowest value within 1.5 × IQR of the lower hinge. No significant ISI-related differences in threshold are evident. *Note.* ISI = interstimulus interval; MEMR = middle-ear muscle reflex.

Figure 1 presents ARTs at 1 and 4 kHz, obtained with ISIs of 2.5 and 8.5 s. In both studies, potential effects of ISI at 1 kHz were assessed via paired Student’s *t* test. In both studies, potential effects of ISI at 4 kHz were assessed via paired sample Wilcoxon test, due to right-skewed distributions of ARTs recorded in the short-ISI measurement condition. In Study 1, no effect of ISI was observed at 1 kHz, (*t*[23] = 1.74, *p* = .09), nor at 4 kHz (*V* = 73, *n*_1_ = *n*_2_ = 24, *p* = .38). Similar results were observed in Study 2, at 1 kHz, (*t*[35] = 0.45, *p* = .66), and at 4 kHz (*V* = 205.5, *n*_1_ = *n*_2_ = 24, *p* = .45).

### Effects of ISI on ART Reliability

**Figure 2.**
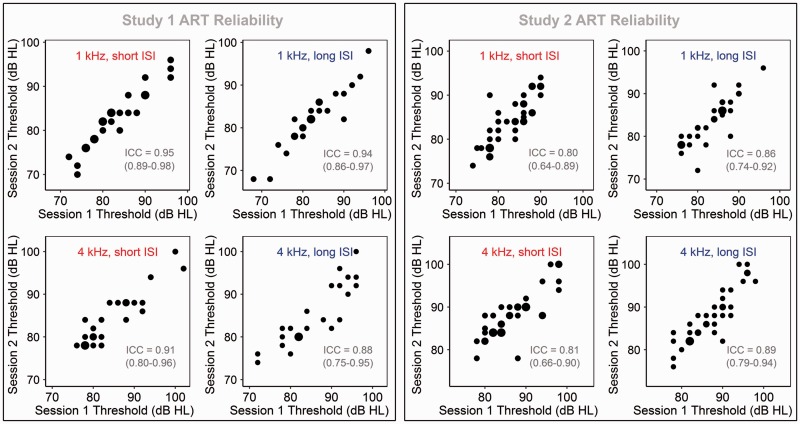
Comparison across test sessions of ARTs obtained in the four measurement conditions, for both Study 1 and Study 2. Point size represents number of observations. ICCs (along with their 95% CIs) are overlaid. *Note*. ART = acoustic reflex threshold; ICC = intraclass correlation coefficient; ISI = interstimulus interval.

Figure 2 presents thresholds at the first and second test sessions for each of the four measurement conditions, with estimated ICCs (including 95% confidence intervals) overlaid. In Study 1, test–retest reliability was excellent for three of the four measurement conditions and good, nearing excellent, for the fourth (4 kHz stimulus, 8.5 s ISI). In Study 2, reliability was good in all four measurement conditions. Because ICCs were consistently high for all of the conditions, no significant effect of ISI could be observed; 95% CIs overlapped substantially. However, it is worth noting that even consistent trends in the hypothesized direction were not evident.

## Discussion

Contrary to our hypotheses, lengthening ISIs from 2.5 to 8.5 s did not significantly reduce 4 kHz ARTs nor improve their reliability. We conclude that elevated clinical ARTs at 4 kHz in young, audiometrically normal people (Gelfand, 2009) are probably not reflective of inadequate recovery of the response between presentations and consequent response fatigue. Of course, findings might be otherwise in alternative populations, especially since ART prevalence is influenced by gender, age, and hearing loss (Flamme, Deiters, Tasko, & Ahroon, 2017). Nonetheless, those clinicians and researchers concerned with the development of guidelines on ART measurement may reasonably conclude that ISIs of 2.5 s are likely adequate, at least in the population sampled here. It is possible that shorter intervals still are implemented by some clinicians; our data cannot speak to their potential effects, whose further investigation might be warranted. Nor can conclusions be drawn on ISIs sufficient for use with wideband measures of acoustic immittance (Feeney et al., 2017) or with prolonged stimuli used to elicit measurable reflex decay (Wilson et al., 1978).

Despite our null results in relation to ISI, it remains possible that elevated 4 kHz ARTs in the studied population are a consequence of some form of across-frequency difference in the dynamic characteristics of the response. Increasing AR stimulus frequency leads to later onset of the response, earlier onset of adaptation, and higher rates of adaptation (Fowler & Wilson, 1984; Jerger et al., 1986; Wilson et al., 1978), some or all of which might play a role in explaining elevated 4 kHz ARTs in human with normal audiograms. This is not to suggest that cochlear synaptopathy plays no role; indeed, one intriguing possibility is that AN pathology impacts ARTs via interactions with dynamic aspects of the response. That is, population variance in synaptopathy might manifest as between-subject variance in the temporal pattern of the reflex, which might in turn impact measured ARTs.

Finally, results confirm those from our previous study (Guest, Munro, Prendergast, & Plack, 2019b) that ARTs can be highly reliable, if consistency is maintained in participant preparation, probe placement, threshold-finding procedure, and other factors influenced by the investigator. This is true even of thresholds obtained with short (2.5 s) ISIs and at relatively high measurement frequencies. ICCs trended slightly lower in Study 2 (conducted by a relatively inexperienced tester) than in Study 1, though CIs overlapped substantially in all but one measurement condition (see Figure 2). Still, the trend suggests a role for practitioner experience in minimizing measurement error.

## Conclusion

The prevalence of elevated 4 kHz thresholds in young, audiometrically normal humans remains unexplained and potentially problematic, especially in the context of investigations of noise-induced auditory pathology. The studies reported here find no evidence that inadequate ISIs are responsible, though other temporal characteristics of ARs at high frequencies might yet play a role. Given growing interest in ARTs as measure of cochlear synaptopathy, more thorough investigation of dynamic response characteristics at high frequencies is perhaps warranted. We have established, at least, that clinically measured ARTs can be highly reliable, if care is taken to ensure consistency of measurement procedures and participant preparation, and that this reliability extends to frequencies as high as 4 kHz. Moreover, the use of relatively short (2.5 s) ISIs appears to be adequate for sensitive and reliable ART measurement, at least in young humans with clinically normal audiograms.
